# Xanthomas Can Be Misdiagnosed and Mistreated in Homozygous Familial Hypercholesterolemia Patients: A Call for Increased Awareness Among Dermatologists and Health Care Practitioners

**DOI:** 10.5334/gh.759

**Published:** 2020-02-28

**Authors:** Fahad Alnouri, Faisal A. Al-Allaf, Mohammad Athar, Zainularifeen Abduljaleel, Moheeb Alabdullah, Dalal Alammari, Menwar Alanazi, Fahmi Alkaf, Abeer Allehyani, Mohammad A. Alotaiby, Abdullah Alshehri, Abdellatif Bouazzaoui, Hussam Karrar, Mohiuddin M. Taher

**Affiliations:** 1 Cardiovascular Prevention Unit, Adult Cardiology Department, Prince Sultan Cardiac Centre, Riyadh, SA; 2 Department of Medical Genetics, Faculty of Medicine, Umm Al-Qura University, Makkah Al Mukarramah, SA; 3 Science and Technology Unit, Umm Al-Qura University, Makkah Al Mukarramah, SA; 4 Molecular Diagnostics Unit, Department of Laboratory and Blood Bank, King Abdullah Medical City, Makkah Al Mukarramah, SA; 5 Department of Dermatology, Prince Sultan Military Medical City, Riyadh, SA

**Keywords:** Familial hypercholesterolemia, Xanthoma, Next-generation sequencing, Mutation, Low-density lipoprotein receptor, Frameshift mutation, Cholesterol, Coronary artery disease

## Abstract

**Background:**

Familial hypercholesterolemia (FH) is an autosomal dominant inherited genetic disorder and results in the development of coronary artery disease (CAD). Clinical diagnosis of homozygous HH patients is usually straightforward because persistent hypercholesterolemia can produce xanthoma and corneal arcus. However, xanthoma may also be misdiagnosed as skin lesions and could therefore be mistreated. The aim of this case study report is to highlight the plight of patients with FH as means of raising awareness of the condition among dermatologists and health care practitioners, also to determine the genotype-phenotype correlation in severely affected homozygous FH proband patients.

**Methods:**

Genetic screening of FH associated genes was performed by Ion Torrent next-generation sequencing and cascade screening by capillary sequencing.

**Results:**

We present two clinical cases with prominent skin lesions seen in a dermatology clinic that were referred to plastic surgery for excision. Genetic testing was performed later, and confirmed common single nucleotide deletion variant (c.2027delG) in the *LDLR* alleles consequent to a frameshift mutation p.(G676Afs*33). In addition to the *LDLR* variant, two possibly damaging *APOB* variants p.(L3313I) and p.(L1212M) and three damaging variants p.(R19*), p.(G83Q) and p.(S474*) in *APOC3, PON2* and *LPL* genes respectively were identified. The *PON2* gene variant p.(G83Q) was found to be novel, while others have been previously reported. Both patients were refractory to pharmacological therapies and are currently on lipoprotein apheresis (LA).

**Conclusions:**

The present report indicates the need for increased awareness of FH, among the public and healthcare practitioners and supports the need for diagnostic screening and cascade genetic testing of this high-risk condition, which could ultimately lead to better prevention of CHD in this lethal condition.

## 1. Introduction

Familial hypercholesterolemia (FH) is a disorder of lipid metabolism characterised by elevated serum levels of total and LDL-cholesterol [1]. If left untreated, FH may cause persistent hypercholesterolemia leading to premature cardiovascular disease [2]. Homozygous FH patients exhibit advanced atherosclerosis and xanthoma in childhood, generally leading to death before the third decade of life. Patients with heterozygous FH generally develop xanthoma in the second decade and, while some patients are affected quite young, most are likely to have a cardiac event before the age of 55 [3]. Because the heterozygous form is asymptomatic, and usually under diagnosed, the majority of patients may not be aware of their illness until they experience a myocardial infarction, which often leads to sudden death or other cardiovascular events [4]. Recent studies on the identification and management of FH patients have suggested that mutation reports should be used to test all first-degree blood relatives to find carriers and offer therapeutic interventions such as statins to reduce lipids levels and decrease the probability of premature cardiovascular disease. FH is most frequently (in over 85% FH cases) triggered by inherited variants in the gene coding for the LDL-receptor (LDL-R). Nonetheless, the range of *LDLR* variants changes between populations with more than 1,700 mutations identified around the world (www.ucl.ac.uk/ldlr), presenting a huge, ongoing challenge in proband identification. There are other identified causes of FH; for example, variations in the apolipoprotein B (ApoB) gene, which encodes the LDL-R ligand, and causes a condition commonly known as familial defective apolipoprotein B (FDB). The other example is the variation in proprotein convertase subtilisin/kexin type 9 (PCSK9) gene or rare variants in the recessive form of the FH-associated LDLRAP1 gene [5].

The frequency of heterozygous FH is usually stated as being ~1:500, but recent studies suggest that the incidence of FH heterozygous can be as high as 1 in ~200–300 [1,6,7]. Similarly, based on these values, the incidence of homozygous FH has now been estimated at 1:160,000–300,000 rather than the historical estimate of 1:1,000,000 [1]. Notably, Saudi Arabia has few reports of prevalence and molecular characteristics of FH [2,8,9,10,11,12]. However, FH is likely to have a higher predominance in Saudi Arabia than in other nations on the basis that consanguineous marriages represent up to 55% of all marriages in the country, as well as absence of either genetic screening programs or national registries for FH [13]. This is confounded by the way that the disease manifests in the patient. The disease is initially asymptomatic and may result in patients not realising they are ill, even if painless xanthomata develop, until a myocardial infarction occurs. By this point, the disease has become hazardous and leads to sudden death or further cardiovascular events.

The clinical manifestations of FH often include tendinous xanthoma, predominantly in the Achilles’ tendons, and in the extensor surfaces of the joints of the elbows, fingers, knees, and toes. Xanthoma may also may manifest as cutaneous xanthoma tuberosum. Tendinous xanthomas may be confused with other growths such as rheumatoid nodules, giant tendon sheath cell tumours, ganglia and sarcomas with xanthomatous changes [14]. In patients with multiple nodules, the disease may resemble Langerhans cell histiocytosis or neurofibromatosis [15]. Additionally present of tuberoeruptive xanthoma and, confusingly, palmar xanthoma, may occur in cases of familial dysbetalipoproteinemia, which is distinct from FH in that it tends to affect triglyceride levels as well as total cholesterol. D dysbetalipoproteinemia is also lethal, with death occurring due to CV events, in common with FH [16].

Histologically, tendinous xanthoma differ from giant cell tumours due to a lack of round cells and a paucity of the multinucleated giant cells [14]. Tendinous xanthoma may also be underdiagnosed and undertreated. Herein, we describe two clinical cases (FN1 and FN4A) with subcutaneous xanthoma, along with our investigation of their first-degree blood relatives.

## 2. Materials and Methods

### 2.1. Study subjects

The analysis was performed in two probands (FN1 and FN4A) and their first-degree blood relatives in the western region of Saudi Arabia. The sample collection and analyses were performed by the Research Ethics Committees at Prince Sultan Cardiac Centre, Riyadh, Saudi Arabia. All subjects gave informed consent.

***History of FN1:*** FN1 is a 20-year old male patient who attended the dermatology clinic at the age of 12 years with complaints of a skin lesion in his right elbow. Subsequently, patient was referred to plastic surgery, where the lesion was removed. The lesion recurred within 4–6 months. His mother coincidentally met a cardiologist and explained the case to him. The cardiologist ran a lipid screen where an abnormal lipid profile was noticed. This led our team to the case. On examination, FN1 had bilateral xanthomas in his elbows and arcus lipoides. The parents were first cousins. The patient was diagnosed (phenotypically) as homozygous FH (HoFH) using the Simon Broome criteria [17]. At the time of examination, FN1 had no evidence of aortic stenosis or aortic regurgitation.

***History of FN4A:*** FN4A is a 29-year old male patient who has attended the dermatology clinic with complaints of a skin lesion in his right elbow. In common with FN1, the patient was subsequently referred to plastic surgery where the lesion was removed. The lesion recurred within 4–6 months, but there was never any suspicion of FH. The patient then developed S-T segment elevation myocardial infarction (STEMI) and was admitted to the Military Hospital in Taif where he underwent a coronary angiogram. He was found to have: 1) Mild left main (LM) ostial lesion, 2) Significant left anterior descending (LAD) mid-segment lesion, 3) Moderate left circumflex (LCX) mid-segment lesion, 4) Right coronary (RCA) ostial tight lesion. He was planned for coronary artery bypass surgery (CABG) but refused and sought a second opinion from our centre (Prince Sultan Cardiac Centre, Riyadh). He had percutaneous coronary intervention (PCI) and stents deployed to the LAD and ostial RCA. The cardiologist conducted a lipid screen in which an abnormal profile was identified. The patient had bilateral xanthomas on his elbows and Achilles’ tendons, and had distal interphalangeal xanthoma in his both hands and feet together with arcus lipoides. The patient was diagnosed phenotypically as homozygous FH (HoFH) using Simon Broome criteria (Scientific Steering Committee, 1991). The parents were first cousins. He had four siblings, all male. Three died of CHD at the ages of 31, 23 and 21 respectively. One brother is alive and has been diagnosed with heterozygous FH (HeFH).

### 2.2. Targeted Ion Torrent PGM Next-generation sequencing (NGS)

NGS was performed in the probands FN1 and FN4A along with two healthy control samples. Genomic DNA isolation, library preparation, enrichment of targeted regions and sequencing were conducted as per the recent report from the Prince Sultan Medical Centre [2]. In brief, we have customized TargetSeq panel for twelve genes implicated in dyslipidaemias (*LDLR, APOB, PCSK9, LDLRAP1, LPL, PON2, ABCA1, APOC2, APOC3, APOA2, ARH* and *APOE*) that also cover exons and flanking intron regions.

Construction of DNA libraries and enrichment of targeted gene regions were achieved using the Ion Plus Fragment Library Kit, Ion Xpress bar-code adapters 1–16 kit and TargetSeq panel (Life Technologies). Emulsion PCR-based template preparation and high throughput sequencing were conducted using the Ion PGM Template OT2 200 Kit and the Ion PGM sequencing 200 kit v2 (Life Technologies) respectively. Samples were loaded onto a 318 sequencing chip for a total of 500 nucleotide flows. Ion Torrent PGM sequencing data were also analysed as per Al-Allaf et al. using CLC Genomics Workbench v8, USA (http://www.clcbio.com) [2]. Additionally, *in silico* functional prediction for nonsynonymous missense variants was determined by SIFT, Provean and PolyPhen [18,19].

### 2.3. Sanger sequencing

Sanger sequencing was conducted to validate the *LDLR* deleterious variant that resulted from NGS analysis and for the screening of the same variant in the first-degree blood relatives. PCR and capillary sequencing were performed as described in our recent report [9].

## 3. Results

### 3.1. Clinical features with xanthoma

**Figure 1:**
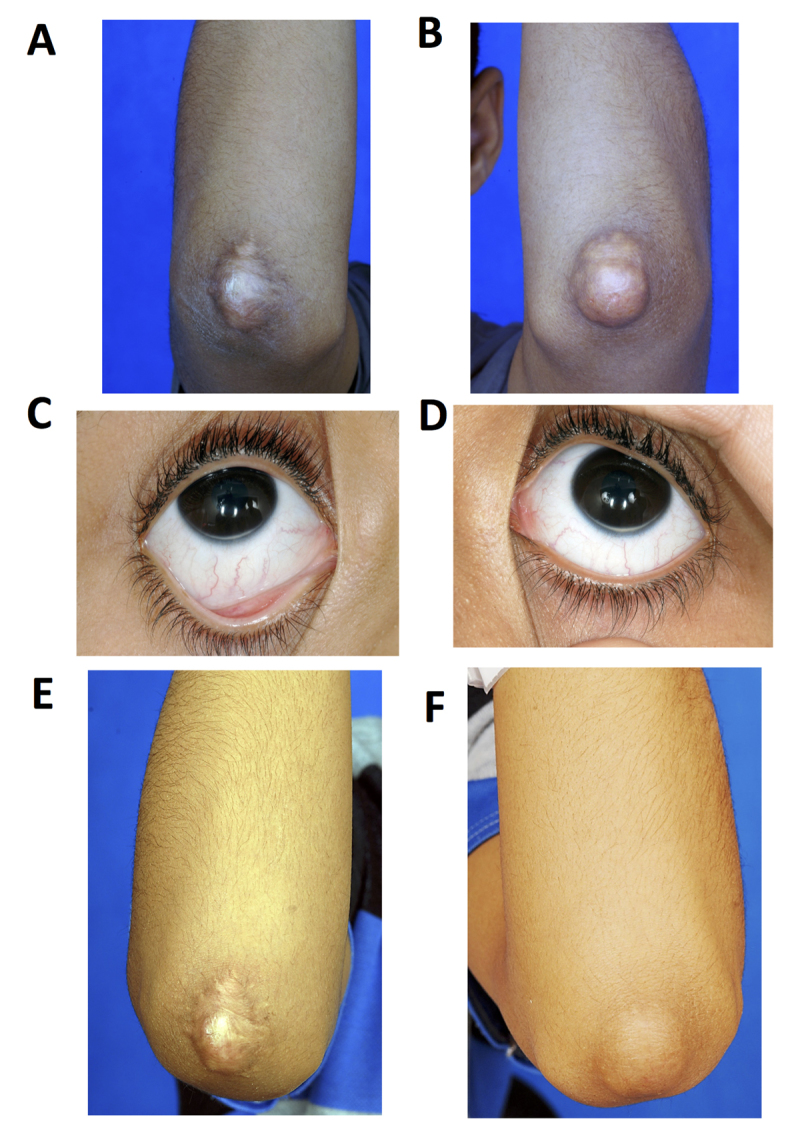
Clinical presentations of patient FN1. Pictures A–D taken when he was 15 years old. Picture E–F taken when he was 18 years old. **A)** Skin xanthoma, right elbow excised. **B)** Xanthoma tendinosum left elbow. **C)** Arcus lipoides, right eye. **D)** Arcus lipoides, left eye. **E)** Skin xanthoma, right elbow disappearance after LDL apheresis. **F)** Skin xanthoma, left elbow disappearance after LDL apheresis.

**Figure 2:**
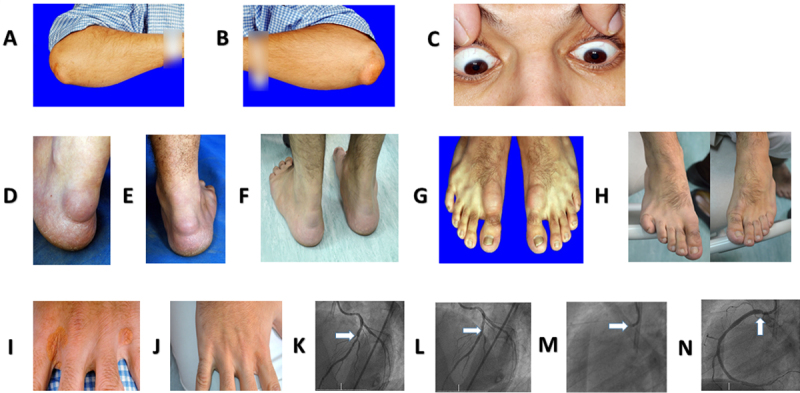
Clinical presentations of patient FN4A. **A)** Xanthoma tendinosum right elbow excised. **B)** Xanthoma tendinosum left elbow. **C)** Arcus lipoides. **D)** Xanthoma tendinosum left foot. **E)** Xanthoma tendinosum right foot. **F)** Xanthoma tendinosum disappearance after LDL apheresis. **G)** Interphalangeal Xanthomas feet. **H)** Interphalangeal Xanthomas feet after LDL apheresis. **I)** Interphalangeal Xanthomas hand. **J)** Interphalangeal Xanthomas hand after LDL apheresis. **K)** Left Anterior Descending (LAD): mid segment significant lesion (white arrow). **L)** Left Anterior Descending (LAD): post Percutaneous Coronary Intervention and stenting (white arrow). **M)** Right Coronary Artery (RCA) totally occluded proximally (white arrow). **N)** Right Coronary Artery (RCA): post Percutaneous Coronary Intervention and stenting (white arrow).

The lipid profile and other clinical features of all the subjects under study are given in Table 1. The off-treatment total and LDL cholesterol levels were considered to be very high compared with the optimal level. TC and LDL-C were as high as 19.12 and 13.1 mmol/l respectively in proband 1 (FN1), and 11.11 and 9.0 mmol/l in proband 2 (FN4A). FN1 had bilateral xanthomas in his elbows and arcus lipoides in his both eyes since the age of 4 years. For lipid lowering, he was taking atorvastatin 80 mg OD and ezetimibe 10 mg OD. Along with the drugs, he was also on biweekly LA. Xanthoma in both elbows regressed after LA (Figure 1). FN4A had bilateral xanthomas in his elbows, Achilles’ tendons, distal interphalangeal xanthomas in the fingers of both hands and the toes of both feet, and arcus lipoides since childhood. He had PCI and stents deployed to his LAD and ostial RCA (Figure 2). In common with FN1, FN4A was receiving atorvastatin 80 mg OD, ezetimibe 10 mg OD and LDL biweekly LA. The skin lesions on both elbows, distal interphalangeal area and Achille’s tendons regressed after LA (Figure 2).

**Table 1:** Off-treatment characteristics and lipid profile of the studied families. The names of families described in this report are anonymised.

Family			FN1 family				FN4A family	

**Family members**	Proband 1	Father	Mother	Sister	Brother	Brother	Proband 2	Brother
**Code**	FN1*	FN1A	FN1B	FN1C	FN1D	FN1E	FN4A*	FN4B
**Sex**	M	M	F	F	M	M	M	M
**Age**	16	51	51	18	14	8	29	36
**Total Cholesterol**	19.12	NA	7.53	6.2	6.84	3.55	11.11	8.57
**Triglyceride**	2.24	NA	1.01	0.48	0.56	0.88	0.54	0.63
**LDL-C**	13.1	NA	5.48	4.1	4.83	1.95	9.0	6.08
**HDL-C**	0.78	NA	1.25	1.29	1.11	1.22	0.81	1.5
**Clinical signs**	Bilateral Xanthomas, Arcus lipoides	No	No	No	No	No	Bilateral Xanthomas, Arcus lipoides, IHD had stents to coronaries	No
**Medication**	LDL apheresis, Atorvastatin, Ezetemibe	Atorvastatin	Atorvastatin	Atorvastatin	Atorvastatin	None	LDL apheresis, Atorvastatin, Ezetemibe	Atorvastatin, Ezetimibe
**Family history of CAD**	Unknown	Unknown	Unknown	Unknown	Unknown	Unknown	Yes	Yes

**Legend:** Normal range for lipid profiles was described in our recent publication (Al-Allaf et al., 2016a). IHD; Ischaemic heart disease. * Index case.

### 3.2. Identification of DNA variants in FH-associated genes by NGS

To detect variants in the 12 FH-associated genes in the probands FN1 and FN4A, CLC Genomics Workbench v8 software (http://www.clcbio.com) was used to map high-quality readouts against a reference human genome (hg19). After mapping, more than 90% of reads were distinctively and cleanly matched to the target area with about 99% of the targeted area covering at least 90% of the mean depth coverage for each analysed sample. The depth of coverage for exons of FN11 and FNA4 samples was 98% and 98.5% respectively, which was adequate to detect variants lying chiefly within the target areas. The identified variants of the 12 tested genes were then compared with the whole-genome sequencing variants available from several SNP reference databases, i.e. dbSNP [20], HapMap [21], Cosmic [22], and Ensemble [23]. The variants identified by Ion Torrent PGM sequencing are shown in Table 2. In the two probands, we identified 13 distinct variants in six genes out of 12 customised genes (Table 2). From the detected all variants, only six variants were found deleterious, and the others were synonymous. We identified only one deleterious variant [c.2027delG, p.(G676Afs*33)] in the *LDLR* gene, which was common in both the probands. Both patients were homozygous for this mutation.

The detected frameshift variant commences a stop signal 33 codons downstream of the deletion, which could possibly lead to a truncated protein that lacks essential functional domains. Other than the *LDLR* variant, two possibly damaging *APOB* variants p.(L3313I) and p.(L1212M) were also found in FN1. In FN4A, three damaging variants p.(R19*), p.(G83Q) and p.(S474*) were identified in *APOC3, PON2* and *LPL* genes, respectively (Table 2). Loss of function variants of *APOC3* can additionally result in hyperalphalipoproteinaemia, which may have an influence on heart disease. The *PON2* gene variant p.(G83Q) was found to be novel while the others are already known. These variants non-*LDLR* may have some contributory effects in enhancing the severity of HoFH in the probands, although with null *LDLR* mutations in both patients, some of these accessory mutations are likely to have little relevance. Our earlier data suggested that the *LDLR* frameshift variant p.(G676Afs*33) could affect the structure and stability of the LDL-R protein, so this variant was validated by Sanger sequencing and further screened in first-degree blood relatives of the probands [9]. This variant p.(G676Afs*33) was confirmed by Sanger sequencing.

### 3.3. Molecular genetic analysis of proband’s family members

**Figure 3:**
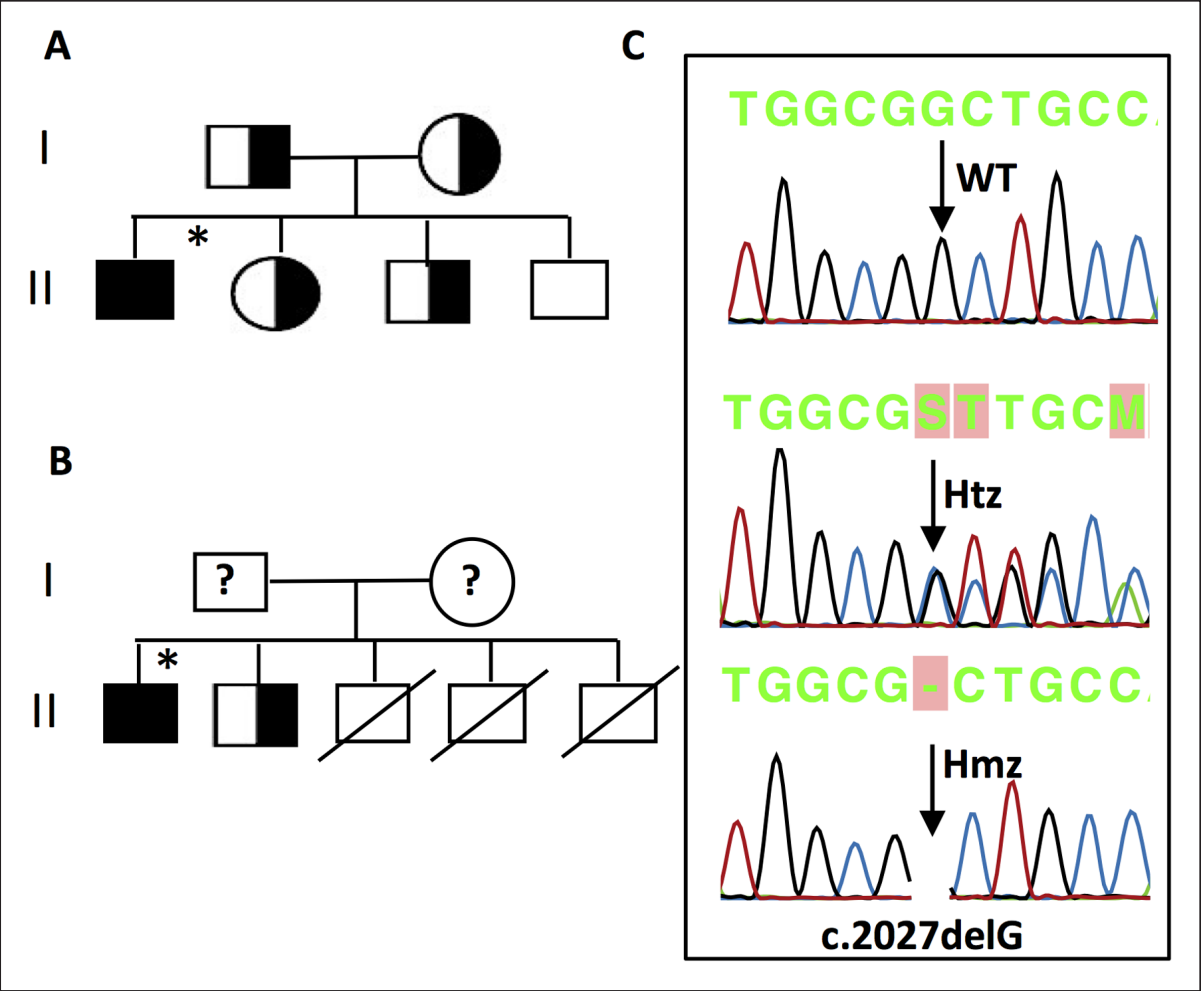
A recurrent frameshift mutation [c.2027delG, p.(G676Afs*33)] identified in exon 14 of the *LDLR* gene. **A)** Pedigree of the families FN1. **B)** Pedigree of the families FN4A. **C)** Representative DNA sequence from a healthy control (Wild type; WT) and heterozygous (Htz) and homozygous (Hmz) of each family. Filled symbols indicate Hmz, half filled indicates Htz, crossed line indicates patients had died and unfilled indicates un-affected individual. ? = NOT available for DNA analysis. * Proband.

In order to screen the first-degree blood relatives of FN1 and FN4A, Sanger sequencing was conducted to identify the *LDLR* variant p.(G676Afs*33). This variant was found in both the probands and in their first-degree blood relatives, all of whom belong to two distinct Saudi families. The probands and their relatives are distributed as follows: six family members of the same family (1 proband + 5 immediate relatives) from one tribe (Nla), who live in the Western regions of Saudi Arabia and two subjects (1 proband + 1 relative) from another tribe (Mla), who also live in the Western region of Saudi Arabia. Figure 3 shows the pedigrees and their representative wild type (WT), heterozygous (Htz) and homozygous (Hmz) DNA sequences in each family. In the FN1 family, the proband was homozygous for the mutation and we found that his parents, one of his brothers and one sister were heterozygous for the same mutation; however, one brother showed no mutation in exon 14 of *LDLR* (Table 1 and Figure 3). In the FN4 family, we observed a homozygous mutation in the proband and a heterozygous mutation in the brother, while samples from the parents were not available for DNA sequencing. Father had died, and their mother lives in a remote area and it is difficult to obtain samples from her.

However, the three brothers were known to have died at the ages of 31, 23 and 21 respectively (Figure 3). They had CABG, but a genetic diagnosis was not made for them. We also sequenced exon 14 of *LDLR* in the 10 control (WT) subjects, i.e. persons with normal lipid profiles (data not shown) and without cardiovascular disease – none had mutations at exon 14 of *LDLR*.

## 4. Discussion

Xanthomas are nodules arising from abnormal deposition of lipid in foam cells and collagen. They occur chiefly in either subcutaneous or tendinous forms depending on location. Joints are usually associated with tendons xanthomas (e.g. on the ankles around the Achillies’ tendon, and on the extensor tendons of the hands), and other areas with subcutaneous types, including planar variants on the dorsum of the hands. Xanthomas develop as a result of leakage of lipids from the vascular tissue into the adjacent tissue, where macrophages subsequently phagocytose these lipids. Cholesterol is not degraded, and accumulates in these cells, making ‘foamy’ macrophages. The extracellular cholesterol crystallises into clefts and stimulates an inflammatory reaction with giant cells and resultant fibrosis [24].

Xanthomas are a classic clinical sign of homozygous FH and are included in international guidelines for clinical diagnosis of the disease. Importantly, xanthomas lead many homozygous FH patients to present in the first instance to dermatologists or plastic surgeons, thereby providing opportunities for prompt diagnosis by physicians aware of HoFH. However, an unusual form of xanthoma may also be misdiagnosed as skin lesions, and could therefore be mistreated. Our patient FN1 had subcutaneous xanthomas on the elbows. FN4A had bilateral xanthomas on the elbows, Achilles’ tendons, and distal interphalangeal xanthomas in his both hands and feet. Interdigital xanthomas are pathognomonic for FH, however there has been a report in which unusual flat interdigital xanthomas in the hands were not associated with FH [25]. Another recent studies on African-American male patients show extensive, multiple xanthomas in areas including the Achilles’ tendon, hands, knees, soles, elbows, and is accompanying with the unusual involvement of the ear [26].

In this study, we found that the patient FN4A has similar unusual xanthomas involving the interdigital webs of the hands. Otherwise, the appearance of the other xanthomas on his body (and also in patient FN1) were found to be typical of HoFH.

Recently, we have reported the mutation p.(G676Afs*33) as a recurrent aberration associated with FH patients among Arab tribes [9,10]. Similarly, in this study we have identified exactly the same frameshift mutation in the probands and their first-degree blood relatives. This mutation was found in the EGF-precursor domain of the LDL-R protein that generates a stop signal 33 codons downstream of the deletion, which would likely result in either degradation of the mRNA or a truncated protein lacking essential functional domains. The segregation pattern of this mutation was compatible with the clinical phenotype of the patients (Table 1). The severity of FH was obviously higher in homozygous subjects than heterozygous subjects (Table 1) due to the deletion frameshift mutation that results in a truncated LDL-R protein or a degraded LDL-R protein, thereby triggering severely restricted LDL-R function. However, the heterozygous patients, with a more moderate FH clinical phenotype, could have one normal allele that delivers some residual LDL-R function.

In addition to *LDLR* causative variants, two variants [p.(L3313I), p.(L1212M)] were also found in *APOB* gene of patient one while in patient 2, three variants p.(R19*), p.(G83Q) and p.(S474*) were identified in *APOC3, PON2* and *LPL* gene, respectively (Table 2). Examination of the association between FH and *PON2* reveals that *PON2* variants are implicated in the clinical presentation of cardiovascular disorder in FH patients [27]. For the *APOB* variants in FN1, these may be of interest to the family, but in patient FN1, these mutations probably have no relevance due to the null mutation in *LDLR*. There are reports that the *LPL* gene variant p.(S474*) can influence the plasma cholesterol and modulate lipid metabolism [28]. Similarly, *APOC3* variants also have an association with hypercholesterolemia [29]. These possibly damaging variants could have some impact on clinical manifestation of FH patients, but again, their influence may be diminished in the face of an *LDLR* null mutation. Lipoprotein (a) values are also elevated in HoFH, and Lp(a) is an independent risk factor for CV events [30]. Therefore, *APOE* genotype has an enormous bearing on Lp(a) levels, so it is logical that *APOE* variants also have a bearing on overall risk in patients with FH.

**Table 2:** Overview of targeted genes variants identified by Ion-torrent PGM.

Samples	Gene	Zygosity	Coding region changes	Amino acid changes	Variant type	Exon	dnSNP	SIFT	Provean	PolyPhen-2

FN1	*LDLR*	Homozygous	c.2027delG	p.(G676Afs*33)	Frameshift	14	Al-Allaf et al., 2016a	n/a	n/a	n/a
	*APOB*	Heterozygous	c.9937C>A	p.(L3313I)	Missense	26	rs146687604	Damaging	Neutral	Possibly damaging
	*APOB*	Heterozygous	c.3634C>A	p.(L1212M)	Missense	23	rs61736761	Tolerated	Neutral	Possibly damaging
	*APOB*	Heterozygous	c.2937C>T	–	coding-synon	19	rs145649470	–	–	–
	*ABCA1*	Heterozygous	c.6183C>T	–	coding-synon	46	rs9282537	–	–	–
	*ABCA1*	Heterozygous	c.3684G>A	–	coding-synon	25	rs2230807	–	–	–
	*ABCA1*	Heterozygous	c.3364C>T	–	coding-synon	23	rs35204915	–	–	–
	*ABCA1*	Homozygous	c.2040C>A	–	coding-synon	15	rs2853579	–	–	–
	*LPL*	Heterozygous	c.207G>A	–	coding-synon	4	rs248	–	–	–
FN4A	*LDLR*	Homozygous	c.2027delG	p.(G676Afs*33)	Frameshift	14	Al-Allaf et al., 2016a	n/a	n/a	n/a
	*ABCA1*	Heterozygous	c.936C>T	–	coding-synon	9	rs2274873	–	–	–
	*APOC3*	Heterozygous	c.55C>T	p.(R19*)	Nonsense	2	rs76353203	n/a	n/a	n/a
	*PON2*	Homozygous	c.248G>A	p.(G83Q)	Missense	4	Novel	Damaged	Deleterious	–
	*LPL*	Heterozygous	c.1421C>G	p.(S474*)	Nonsense	9	rs328	n/a	n/a	n/a

**Legend:** n/a: not applicable.

In conclusion, we identified a common, life-threatening frameshift mutation in the *LDLR* gene in two Arab HoFH patients with xanthoma, both of whom were originally referred to dermatologist and treated surgically by plastic surgeons. This experience indicates that there is an urgent need for increased awareness of FH, among the public and healthcare practitioners, and additional support for diagnostic and cascade screening of this high-risk condition. Accurate diagnosis and early intervention in HoFH have the potential to retard progression of life-threatening CHD. We urge our general practice, dermatology and plastic surgeon colleagues to consider the possibility of HoFH in patients presenting with clinical signs similar to those described in our probands.
